# Communicating information on nature-related topics: Preferred information channels and trust in sources

**DOI:** 10.1371/journal.pone.0209013

**Published:** 2018-12-12

**Authors:** Emily J. Wilkins, Holly M. Miller, Elizabeth Tilak, Rudy M. Schuster

**Affiliations:** 1 Independent Contractor: Contracted to the U.S. Geological Survey, Fort Collins, Colorado, United States of America; 2 Fort Collins Science Center, U.S. Geological Survey, Fort Collins, Colorado, United States of America; 3 Department of Journalism and Media Communication, Colorado State University, Fort Collins, Colorado, United States of America; Indiana University Bloomington, UNITED STATES

## Abstract

How information is communicated influences the public’s environmental perceptions and behaviors. Information channels and sources both play an important role in the dissemination of information. Trust in a source is often used as a proxy for whether a particular piece of information is credible. To determine preferences for information channels and trust in various sources for information on nature-related topics, a mail-out survey was sent to randomly selected U.S. addresses (*n* = 1,030). Diverse groups of people may have differing communication preferences. Therefore, we explored differences in channel preferences and trust by demographics using regression models. Overall, the most preferred channels were personal experience, reading online content, and watching visual media online. The most trusted sources were science organizations, universities, and friends/family. Channel preferences varied the most by education level and age, while source trust was most influenced by education, race, age, and size of current residence (rural-urban). The influence of demographics varied depending on the individual channel and source, with some groups preferring certain channels or sources but not others. Results are useful to consider when disseminating information on nature-related topics to a general public audience. More broadly, results also suggest spreading information using different channels and sources depending on the specific audience being targeted.

## Introduction

Communication methods alter perceptions of environmental issues and ultimately behavior [1]. Scientists, environmental advocates, and natural resource managers are just a few groups who frequently communicate nature-related information to the general public. Environmental communication efforts can affect anything from consumers buying sustainable products, to hikers staying on trail to prevent erosion. Therefore, it is important to understand ways to better communicate environmental information to the general public [1].

Two main factors to consider when disseminating information are: which channel should be used to disperse the information, and which source is providing the information. Sources are the organization or individual who creates a message, while channels refer to the methods through which the information is communicated to someone [2]. Both source preference and channel preference are highly individualized; there can be differential effects of trust dependent upon the various domains. Yet trust can greatly impact how an individual cognitively processes and accepts communicated messages [3]. Understanding trust in sources and channel preferences can help communicate messages more effectively.

The overall goal of this study is to better understand how to effectively communicate information on nature-related topics, such as recreational activities, wildlife, natural areas, or conservation issues, to the general public. The objectives of this study are to: (1) determine the public’s most preferred channels for receiving information on nature-related topics, (2) investigate which sources have high levels of trust on nature-related topics, and (3) explore differences in channel preferences and levels of trust based on demographics.

### Channels of information

Information channels refer to the places someone goes to obtain information (i.e., online, radio, visual media, taking with others). This can also be referred to as the mode in which the message is delivered. Preferences for information channels change over time as new means of communication are developed and become mainstream. People do not always acquire information from their preferred channel because sometimes other channels of information are more accessible [4, 5]. However, organizations may have more success disseminating information if they communicate through preferred channels.

The uses and gratifications theory states that individuals prefer and use different channels of communication to gratify their needs [6]. Therefore, preferences for information channels can vary based on many factors, such as demographics or location. In the United States in 2016, television (TV) was the most popular channel for obtaining news information, followed by the online channel. However, there were substantial differences in channel use depending on age. Older people were more likely to obtain their news from print newspapers and TV, while younger people were more likely to gather news using an online channel [4]. Research on communicating water resources information revealed that older people (ages 65+) and those with less education were more likely to want to watch TV to seek the information, while younger people (ages 18–44) and those with more education were more likely to seek water resource information by visiting websites [7]. In addition, channel preferences were impacted by community size, with people in urban areas more likely to want to visit websites than people in small towns or rural areas [7]. Race, ethnicity, and gender can also impact preferences for specific channels [8].

Additionally, the type of information being sought can impact the channel used to obtain that information. Research suggests that individuals largely do not prefer listening to news information sources [4]. However, research regarding disaster response communications has shown that listening (e.g., radio) has a higher preference in this news form as it was rated second most preferred source behind TV [9]. Conversely, online was found to be the favorite way to obtain information on science issues [10]. Preference can also vary depending on how great the information need is. When information need is high (high uncertainty), people prefer to use others they know and prefer oral communication over written information [11]. Therefore, understanding preferences for information channels on one topic may not carry over to other topics. Although these studies have investigated channel preferences by demographics, none have focused on channel preferences for receiving nature-related information specifically.

Channels that have relatively low usage may still have important impacts if they are used by organizations that have high credibility. Additionally, a channel may not be preferred by the overall population, but could be an important communication means for a certain demographic audience. In summary, the body of literature suggests that to maximize message effectiveness, it is beneficial to identify groups that may prefer specific channels, coupled with sources they perceive as trustworthy.

### Trust in sources

The source of information refers to the person or organization that is distributing the information [12, 13]. Trustworthiness and expertise are two key characteristics that are associated with the believability of the source. Expertise refers to whether the communicator has sufficient knowledge of the issue, whereas trust is related to whether the communicator will be likely to tell the truth. Both expertise and trust aid in the judgments a receiver makes of the message being communicated [14, 15].

As a multi-faceted concept, trust includes cognitive, emotional, and behavioral dimensions that combine into one collective event that informs an individual [16]. The initial cognitive process occurs in order to discriminate or judge the trustworthiness of the message [16]. In the literature, trust has been defined both as synonymous with credibility or as a function of credibility. When credibility of the message source is high, it can positively impact the trust that one has in that source. Likewise, when the credibility of the source is low, it can undermine the degree of trust one would hold for that source.

Trust in sources helps to determine how likely it is that someone will accept the information being presented. This can also impact how likely they are to change behavior based on the information [17]. Rather than do extensive research to verify all of the details in the information, people tend to judge the truthfulness of information based on how credible they believe the source is [18, 19]. For example, people who trust scientists on environmental issues are more likely to believe their message on climate change [20]. Oftentimes, if people do not trust the origin of information, they have a difficult time accepting and trusting the information presented, even if it is credible [21, 22]. Similarly, others suggest that trust in the source is needed for the public to take action in crisis situations [23]. Essentially, the degree of trust is a cue toward efficiently evaluating information [24].

As with preferred channels of information, trust in sources can also vary. Factors that impact trust can include time [25, 26], demographics [3, 27, 28], location [29], or specific topic [30, 31]. Trust in both human sources and institutions has declined in the U.S. from 1972–2012 [25]. The authors also found that Americans of all ages and generations reported declines in trust over time, and that trust is lower when inequality is high [25].

Overall trust in national news sources by younger individuals (age 18–49) is less than that by older individuals (ages 50+). However, when it comes to their source of choice, trust levels are similar for all ages [4]. While the degree of trust is similar, younger people appear to be more selective about which sources of information they trust. There has been a substantial amount of research on the association of specific demographic factors and level of trust in scientific communications [32–36]; however, there is less research on source trust when disseminating information on other topics. Using linear regression models, one study found that more education predicts greater trust in science, while women and people of color tend to have lower trust in science [36]. A different study found that older people and African Americans were less likely to trust scientists on environmental issues [3]. Trust in the news media was the most influenced by demographics, with females and African Americans more likely to trust the news media, and those with higher education levels and incomes less likely to trust the news media [3].

The degree of trust in sources can impact perceptions of environmental issues [20, 37] and thus has many consequences and implications. In a U.S survey in 2006–2007, only 32% of people stated they trust scientists on environmental issues either “completely” or “a lot” [38]. In the United Kingdom, survey results revealed that citizens had the highest level of trust in university scientists and environmental organizations, while government sources yielded the lowest degree of trust for environmental issues [39]. Understanding trust in sources is insightful for selecting the best messenger to communicate information that is likely to be accepted.

### Theoretical background

The Elaboration Likelihood Model (ELM) is a dual-process theory that provides theoretical explanation of information processing in the formation of judgments or attitudes [19]. When presented with a persuasive argument, cognitive mental processes ensue in order to assess (i.e., evaluate and judge) the argument. Petty and Cacioppo have defined two distinct routes of processing a person can engage in when presented with a persuasive argument: the central route and the peripheral route [19]. The central route involves a greater need for cognitive effort in the elaboration of an argument and involves analyzing the information in an effortful manner. The peripheral route, on the other hand, uses cues in order to formulate a judgment. When motivation and ability to fully analyze a message are high, the central route of judgment formation will ensue. Conversely, when motivation and ability to process persuasive information are low, a person will use cues or shortcuts in order to assess information and form judgments. Therefore, according the theory, yielding to a persuasive argument is partially dependent upon individual factors (i.e., motivation, knowledge and experience) as well as the manner in which people process that argument.

There are a number of heuristics or cues that can impact an individual’s attitude formation. One such cue is the construct of credibility or trust in the message source [19]. Trust in the message source can enhance an ability to support a persuasive argument. Several source cues have been identified, including perceived similarity to the individual, symbols of authority (e.g., education, organization type), style of speech, humor, physical attractiveness, familiarity, caring, and technology-based (e.g. websites), among others [40, 41]. Depending on the communication channel, these factors may be exhibited differently, yet all add to the process of judging the trustworthiness of a message source.

A receiver’s judgment of the credibility or trustworthiness of a message source is a key component to the evaluation of a persuasive message very early in that process [19]. Understanding which sources of environmental messages are considered trustworthy by receivers can facilitate positive processing of information and is vital in effective communication planning.

## Methodology

### Survey instrument

This research was a piece of a broader effort to inform the 2018 update of the North American Waterfowl Management Plan. The instrument consisted of four sections: nature and wetlands activities, channels and sources of information about conservation issues, opinions about wetlands, and demographics. For this paper, the focus is on the questions pertaining to channels and sources of information and demographics. Respondents were asked to rate their preferences for information channels when looking for information about nature-related topics, such as recreational activities, wildlife, natural areas, or conservation issues. Ten channels were listed (see the full list in the results section for the exact wording given in the survey), and respondents were asked their preference on a 4-point Likert scale from “not at all preferred” (0) to “very preferred” (3). Following this, ten information sources were listed (see the full list in the results section for the exact wording given in the survey), and respondents were asked to rate their levels of trust when looking for information about nature-related topics, on a 5-point Likert scale from “do not trust at all” (0) to “trust completely” (4).

The sources and channels listed were modified from those on the Canadian Nature Survey. However, the Canadian survey asked for where people most often obtain information, whereas our survey instrument asked for preferences and levels of trust [42]. Following their survey instrument, we also had an “other” option for both channels and sources, which respondents could fill in if desired. Due to low response in the “other” categories (*n* = 15 for other channels, *n* = 21 for other trust in sources), these data were not analyzed. There was also an option on the survey to check a box saying “I do not look for information about nature-related topics,” and instructions to skip both questions. Therefore, people who do not seek information on nature-related topics are not included in the analysis on preferred channels and trust in sources. Demographics collected include: age, gender, education level, population size of current residence (on a 5-point scale, from rural to large urban), population size of childhood residence (on a 5-point scale, from rural to large urban), ethnicity, and race.

To assess for other potential differences between those who look for information on nature-related topics and those who do not, additional questions were asked on outdoor recreation participation, wetlands visitation (within the last 12 months), and knowledge of wetlands in their community. The complete survey instrument can be found in the supporting information (S1 File).

### Data collection

Data were collected by mailing the survey to 5,000 randomly selected adults in the United States from January-March 2017. The sample was purchased from Survey Sampling International to reflect the demographics of the U.S., and addresses were chosen in proportion to the population of each state. The survey was implemented using the tailored design method, so all respondents received a follow-up reminder postcard, and those who still had not responded received a replacement survey [43]. Additionally, those who never responded received a short questionnaire to assess non-response bias. Therefore, each mailing address could be contacted up to four times. There was no incentive for completing the survey.

This study was approved by the U.S. Office of Management and Budget (OMB Control #1028–0120). We did not get explicit written or oral consent from respondents, but sent a letter with the survey stating that participation was voluntary, and that names and addresses would be deleted from the mailing list and never connected to answers in any way. Mailing a survey back implied consent. A database with all the raw data, minus any personal identifiers, can be found at https://doi.org/10.5066/F7G15ZQ6 [44].

### Data analysis

Data were analyzed using SPSS version 24. Frequencies of responses and means are presented to better understand overall preferences for channels of information and levels of trust in sources on nature-related topics. Data were then analyzed to understand differences in preferences and trust by demographics. Principle component analysis was used to reduce the 10 channels and 10 sources into a smaller set of variables to use as dependent variables in regression models, as was done by others when modeling preferences for information sources [45]. Principle component analyses were run using varimax rotation and Kaiser Normalization. The number of components was chosen based on eigenvalues greater than 0.5, which was selected with the intention of obtaining greater than three components per analysis. Internal consistencies for the collapsed categories were tested using Cronbach’s alpha (used for categories with three items) and Spearman-Brown’s predicted reliability (used for categories with two items) [46].

To explore the impact of demographics, stepwise linear regressions were run with the collapsed channels and sources as the dependent variables, and demographics as the predictors. Demographics in the models were: age, gender, education, population size of current residence, population size of childhood residence, race, and ethnicity. Due to the small number of minority respondents, race was collapsed to “people of color” and “white.” Ethnicity is also on a dichotomous scale, either “Hispanic or Latino” or “Not Hispanic or Latino.” For each regression, the standardized coefficients from the predictors in the best fitting model are presented.

### Response and weighting

The response rate was 23.4%; 1030 surveys were returned completed, and 595 were undeliverable. Of those who did not complete the survey, 275 sent back the non-response questionnaire. This survey did have some selection bias. Those who responded to the full survey were more likely to participate in all nature-related activities (p<0.05 for 7/10 activities listed), visit wetlands (χ^2^ = 41.233, df = 1, p<0.001), and have knowledge of wetlands in their communities (χ^2^ = 42.886, df = 2, p<0.001) than those who responded to the non-response survey. This indicates that our sample is overall more nature-oriented than the general U.S. public. However, this paper is primarily interested in the preferences of those who actually do look for information on nature-related topics. Understanding channel and source preferences for people who do not want or need the information would not be useful. If the sample did not have selection bias, more respondents would have likely checked that they do not look for information on the topic rather than answer the question. Therefore, the selection bias towards nature-oriented people is likely not an issue for this paper since they are the ones most likely to be seeking information on nature-related topics.

However, compared to the Census, the sample is not demographically representative of the U.S. population, which could influence results. Data presented for general results were therefore weighted using Census data for both gender and age (Table 1) since there was a higher response from males and older people than would be expected from the population. We used the post-stratification method of cell weighting because it is a common technique used in survey research to make the sample more similar to the population in some way, when characteristics of the population are known [47–49]. Although weighting generally makes the results more representative of the population, it does increase the variance of the estimates [48]. Both age and gender have been shown to affect channel preference and trust in sources [e.g. 3, 4]; therefore, both were applied as weights to make the results more representative of the general population. Weights were not applied for the regression analyses since the aim was to investigate differences by demographics.

**Table 1:** Weights applied to each respondent’s answers based on gender and age.

Age	Male	Female
**18–44**	1.952	2.550
**45–64**	0.563	1.095
**65 +**	0.343	1.097

The sample also showed differences between the survey respondents and the general public for other demographics, including level of education, ethnicity, and race. However, with the addition of each variable for weighting, the sample size for each grouping decreases [47]. Weighting with small numbers in each category gives a large voice to a small handful of people who may not be representative of their demographic. Therefore, the data was only weighted on gender and age to maintain a reasonable number of respondents per category. Being unable to weight by other demographic variables leaves the possibility that the preferences of those who are underrepresented in this sample are not accurately portrayed in this analysis.

## Results

### Profile of the respondents

This sample had respondents from 49 U.S. states and is geographically diverse within the U.S. However, the demographics overall were not representative of the general U.S. population, with higher representation in this sample from males, older people, highly educated people, and white people (Table 2). Although there also appears to be major differences among the current residence statistics, this is likely because our survey instrument asked people to identify their perception of where they lived, which is important as it may relate to their communication preferences. Since data were weighted for gender and age, unrepresentativeness in these categories does not need to be considered when interpreting the data.

**Table 2:** Demographics from the survey sample compared to the U.S. census bureau.

	Sample (%)	Census (%)
*Gender*		
Male	65.1	49.2
Female	34.9	50.8
*Age*		
18–44 (% of adults)	21.4	48.1
45–65 (% of adults)	45.8	34.7
65+ (% of adults)	32.7	17.2
*Education*		
High school degree or less	17.4	41.1
Some college or Associates	30.3	26.4
Bachelor's degree	26.8	20.5
Graduate degree	25.5	12.0
*Current residence*		
Urban (50,000+)	46.8	71.2
Urban cluster (2,500–50,000)	37.9	9.5
Rural (<2,500)	15.4	19.3
*Childhood residence*		
Urban (50,000 +)	44.7	N/A
Urban cluster (2,500–50,000)	36.9	N/A
Rural (< 2,500)	18.4	N/A
*Ethnicity*		
Hispanic	5.6	17.1
Not Hispanic	94.4	82.9
*Race*		
People of color	13.9	26.4
White	86.1	73.6

Just under two-fifths of people indicated that they do not look for information on nature-related topics (190/1012). There were no significant differences between those who do look for information on nature-related topics and those who do not in terms of gender, urban-rural residence, ethnicity, or race. However, there were differences in age and education, with people 65+ less likely to look for information on nature-related topics (χ^2^ = 18.044, df = 2, p<0.001, Cramer’s V = 0.135) and people with more education more likely to look for information (χ^2^ = 11.566, df = 3, p = 0.009, Cramer’s V = 0.107). Those who do not look for information on nature-related topics were significantly less likely (p<0.05) to participate in all 10 listed outdoor recreational activities within the last 12 months than those who do look for information on nature-related topics.

### Channels of information

Overall, the strongest preference for receiving information on nature-related topics was through personal experience, followed closely by watching visual media through cable/satellite/network, and watching visual media online (Table 3). The least preferred channels were listening to information (recorded or live audio media) and attending educational opportunities. For example, 38% of people said personal experience was a very preferred channel, compared to only 4% who stated listening to recorded audio media was a very preferred channel.

**Table 3:** Preferences for channels of information on nature-related topics.

Channel	Not at all preferred (%)	Slightly preferred (%)	Somewhat preferred (%)	Very preferred (%)	Mean
Receive or follow **online** communications (email updates or newsletters, social media, etc.)	31.1	24.2	25.1	19.6	1.33
Read or access **online** content (websites, apps, blogs, magazines, newspapers, books, reports, etc.)	15.7	16.9	33.6	33.8	1.85
Read **printed** publications (magazines, newspapers, books, reports, newsletters, brochures, etc.)	16.6	27.5	34.4	21.5	1.61
Watch visual media **online** (videos, webinars, television shows, movies etc.)	12.8	22.1	34.8	30.3	1.83
Watch visual media through cable, satellite, or network (television shows, movies, etc.)	15.5	21.0	34.7	28.9	1.77
Listen to **recorded** audio media (podcasts, audio books, etc.)	62.5	24.1	9.2	4.2	0.55
Listen to **live** audio media (radio, etc.)	47.3	30.2	16.5	6.0	0.81
Talk with other people about nature-related topics (friends, family, colleagues, etc.)	15.3	24.6	36.2	23.8	1.68
Through personal experience	13.8	14.9	33.1	38.1	1.96
Attend educational opportunities (courses, seminars, conferences, etc.)	51.7	26.7	12.9	8.8	0.79

Mean is on a scale from 0 (not at all preferred) to 3 (very preferred). *n* = 803–850.

After running a principle component analysis, the 10 channels were collapsed into 6 groups (Table 4). Receive/follow online communications and read/access online content were collapsed to “online”; watch visual media online and watch visual media through cable were collapsed to “visual”; listen to recorded audio media and listen to live audio media were collapsed to “audio”; and talk with other people and through personal experience were collapsed to “personal exp/com.” Reading printed publications and attending educational opportunities were both kept separate since they did not fit with the other items. Although listening to recorded audio media loaded on two components, it was combined with listening to live audio media, as the loading was higher.

**Table 4:** Principle component analysis for channels of information.

	Components					
Channel	1	2	3	4	5	6
Receive or follow online communications		.874				
Read or access online content		.807				
Read printed publications						.881
Watch visual media online			.837			
Watch visual media through cable, satellite, or network			.870			
Listen to recorded audio media				.759	.437	
Listen to live audio media				.878		
Talk with other people about nature-related topics	.840					
Through personal experience	.836					
Attend educational opportunities					.860	
**Reliability** (Spearman-Brown)	.808	.753	.774	.706	-	-

Varimax rotation. Values <0.4 are not presented.

Overall, education was the most influential demographic for channel preference, since it was a significant predictor for all channels (Table 5). Those who were more educated were more likely to want to receive information on nature-related topics from all channels. In fact, education was the greatest predictor for online, audio, personal experience/communication, and educational opportunity preferences. Age was the most influential predictor of visual preferences, while childhood residence was the most influential predictor of printed information preference.

**Table 5:** Preferences for channels of information on nature-related topics by demographics. Numbers represent standardized beta coefficients from stepwise regressions.

	Online	Visual	Audio	Printed	Personal Exp/Com.	Educational Opp.
	(*n* = 858)	(*n* = 864)	(*n* = 858)	(*n* = 860)	(*n* = 862)	(*n* = 852)
Age	-0.158***	0.076*	-	0.106**	-0.124**	-
Gender	-	-	-	-	-	0.071*
Education	0.182***	0.074*	0.075*	0.075*	0.137***	0.187***
Current residence	-	-	-	-	-	-
Childhood residence	-	-	-	-0.129***	-0.128***	-
Race	-0.082*	-	-	-	-0.085*	-
Ethnicity	-	-	-	-	-	-
**Adjusted R**^**2**^	0.063	0.008	0.004	0.028	0.050	0.037

* = p< 0.05

** = p< 0.01

*** = p< 0.001

Dichotomous codes: Gender: M (1), F (2); Race: white (1), POC (2); Eth.: Hispanic (1), non-Hispanic (2)

Residence is on a scale from: rural (1) to large urban (5)

Online media had higher preference from younger people and white people, while visual media had higher preference from older people. Audio media preferences were only impacted by education. Older people and those raised in more rural areas were more likely to prefer printed publications. Personal experience and communication were preferred by younger people, people raised in more rural areas, and white people. Finally, women were more likely to prefer educational opportunities. Both ethnicity and the size of current residence (urban-rural) did not impact any channel preferences. For these six regressions, the adjusted R^2^ ranges from 0.004 (audio preference) to 0.063 (online preference).

### Trust in sources

Respondents had the highest levels of trust in scientific organizations for nature-related topics, followed closely by universities/educational organizations and family/friends (Table 6). People had the lowest trust in religious organizations, the national media/news, and the local media/news. A majority of people (64%) trusted scientific organizations either a lot or completely, compared to 19% that trusted religious organizations a lot or completely for information on nature-related topics.

**Table 6:** Trust in sources for information on nature-related topics.

Source	Trust little/ not at all (%)	Trust somewhat (%)	Trust a lot/ completely (%)	Mean
Federal government	29.0	35.4	35.6	2.05
State government	22.4	35.8	41.8	2.21
Local government (city, county, etc.)	18.4	37.2	44.4	2.32
Conservation groups	13.5	32.2	54.3	2.55
Universities/Educational organizations	12.8	24.8	62.4	2.65
National media/news	43.4	39.2	17.4	1.59
Local media/news	35.4	42.9	21.7	1.78
Friends, family, neighbors, colleagues	10.6	30.5	58.9	2.64
Scientific organizations	11.1	24.0	64.0	2.77
Religious organizations	50.9	30.2	18.9	1.47

Mean is on a scale from 0 (do not trust at all) to 4 (trust completely). *n* = 868–880.

The 10 sources were collapsed to 5 based on the principle components analysis (Table 7). Federal government, state government, and local government were all collapsed to “government”; conservation groups, universities, and scientific organizations were collapsed to “uni/sci/cons”; and national media/news and local media/news were collapsed to “news.” Friends/family and religious organizations were both kept separate since they were not highly associated with any of the other items.

**Table 7:** Principle component analysis for trust in sources.

	Components				
Source	1	2	3	4	5
Federal government	.847				
State government	.913				
Local government (city, county, etc.)	.830				
Conservation groups		.834			
Universities/Educational organizations		.823			
National media/news			.854		
Local media/news			.881		
Friends, family, neighbors, colleagues				.958	
Scientific organizations		.826			
Religious organizations					.974
**Reliability (**1 & 2: Cronbach’s alpha; 3: Spearman-Brown)	.896	.868	.881	-	-

Varimax rotation. Values <0.4 are not presented.

Every demographic variable was significantly influential in predicting trust for at least one source (Table 8). For the government, older people, more educated people, people in urban areas, and Hispanic/Latino people were more likely to express trust, with education being the most influential predictor. Women, more educated people, those in urban areas, and white people were more likely to trust universities/science organizations/conservation organizations. Again, education was the most influential predictor. Many demographics were influential for trust in the news, with older people, females, higher educated, urbanites, and people of color more likely to trust the news; gender was the greatest predictor. The only two predictors of trust in family/friends were childhood residence and race, with those growing up in more rural areas and white people more likely to trust family/friends. Finally, older people, people with less education, racial minorities, and Hispanic/Latino were more likely to trust religious organizations. The greatest predictor for trust in family and friends was race, while the greatest predictor for trust in religious organizations was age. For these five regressions, the adjusted R^2^ ranged from 0.019 (predicting trust in family/friends) to 0.086 (predicting trust in universities/science/conservation organizations).

**Table 8:** Trust in sources for information on nature-related topics by demographics. Numbers represent standardized beta coefficients from stepwise regressions.

	Gov.	Uni/Sci/ Cons.	News	Family/ Friends	Religion
	(*n* = 886)	(*n* = 888)	(*n* = 883)	(*n* = 886)	(*n* = 871)
Age	0.069*	-	0.149***	-	0.186***
Gender	-	0.165***	0.155***	-	-
Education	0.184***	0.214***	0.129***	-	-0.073*
Current residence	0.101**	0.075*	0.088*	-	-
Childhood residence	-	-	-	-0.078*	-
Race	-	-0.078*	0.088*	-0.116**	0.076**
Ethnicity	-0.076*	-	-	-	-0.101***
**Adjusted R**^**2**^	0.052	0.086	0.068	0.019	0.051

* = p< 0.05

** = p< 0.01

*** = p< 0.001

Dichotomous codes: Gender: M (1), F (2); Race: white (1), POC (2); Eth.: Hispanic (1), non-Hispanic (2); Residence is on a scale from: rural (1) to large urban (5)

## Discussion

The results of this study can contribute to effectively communicating information to the public on nature-related topics, such as conservation, outdoor recreation, natural resources, public lands, or wildlife. The most preferred channel for our sample is personal experience, so it is important that protected areas and nature organizations continue offering programming where people gain hands-on experiences. However, although personal experience was the most preferred channel, attending educational opportunities (i.e. courses, seminars, and conferences) was one of the least preferred channels in this sample. High preference for personal experience but low preference for educational opportunities implies that the personal experience must be more interactive, hands-on, or conversational to be preferred, and should not be advertised as an educational opportunity. Additionally, the subsequent preferred communication channels are reading online content and watching visual media online. Thus, organizations could benefit from focusing on their online presence and making sure information is available online, preferably as both text and video content, if applicable. There was overall low preference for audio media, both radio and podcasts, so it might not be worth the time to create audio media when trying to disseminate information to a general audience.

Trust in sources is important because people tend to use trust as one way to assess whether or not they should accept the information being presented [18, 19]. As individuals process information, trust in sources is used as a heuristic, which helps to alleviate uncertainty in processing. Using trust in this manner creates a more efficient means in which to evaluate environmental information [3]. Within our study, the most trusted source identified was science organizations, followed by universities, and friends/family were the third most trusted source. To increase the perceived credibility of information, it might be beneficial to partner with a science organization or university to disseminate information, at least for our survey population. In addition, people had high trust in their friends/family/neighbors. One way to tap into this would be to post information on social media, which would give people the opportunity to easily share information within their network. Hearing information from their network may allow yielding to nature-related messages more easily, particularly for individuals that have less trust in a scientific organization. However, the source of the social media post would still likely matter, and more people would trust it coming from science or universities rather than general media outlets.

Compared to surveys in 2006 and 2007 which found only 32% of people trusted scientists on environmental issues either “completely” or “a lot” [50] this survey found 64% trust science organizations either “completely” or “a lot.” Our finding of greater trust in scientists may be because the only people asked to answer our survey questions on trust were those who do look for information on nature-related topics. Therefore, our sample is likely more environmentally-minded than the general public. Additionally, the heightened trust in scientists may be attributed to the fact that this sample is higher educated than the general public, and the results showed that those with more education were more likely to trust scientists/universities/conservation organizations. The larger trust in scientists found in our sample can also be explained by the significance of the source cue for the individual. Familiarity and similarity of the source, among other factors, can aid in the degree of perceived trustworthiness of the source [40, 41]. In our population, findings suggest that trust in scientific organizations enhances a likely acceptance of nature-related communications. For a smaller percentage of our population that lack trust in scientists, this would negatively impact information processing of nature-related communications from this source.

There were also some interesting differences in communication preferences by demographics. Older people were more likely to prefer visual and printed communication, and younger people were more likely to prefer online communication and personal communication or experience. Our findings on channel preference differences between age groups concurs with others’ findings that, for information on water resources, older people were more likely to want to receive information through the TV, and younger people are more likely to want to prefer getting information online [7]. These results also align with another survey that found older people are more likely to consume news from print papers, while younger people are more likely to consume news online and value personal communication [4]. Despite differing information topics studied, some of the general trends were the same (e.g., younger people having a stronger preference for online information). Although there may be some general trends common among many topics, people do have different channel preference and trust in sources depending on the topic of the information disseminated [6, 9, 10]. Therefore, we do not recommend extrapolating our results on preferences for communicating nature-related topics to disseminating information on other topics.

For differences in trust, we found that women, urbanites, white people, and highly educated people were more likely to trust universities/science/conservation organizations. Another study found that white people and those with more education were more likely to trust science in general; however, the researcher found the opposite for gender, in that women were less likely to trust science [36]. The difference may be because, after conducting a principle component analysis, we combined science organizations with universities and conservation organizations. The differing results could also be because people may trust science organizations for information on nature-related topics but not necessarily other topics. The demographic analyses also fit with previous findings that women were more likely to trust the news for environmental information [3]. However, we found that those with more education were more likely to trust news sources as well. Our regressions also support previous results that young people are less likely to trust the news in general [4]. Understanding preferences by demographics could be useful when trying to spread nature-related information to a specific group of people. Results broadly suggest it would be beneficial to disseminate information using different channels and sources depending on the specific audience being targeted.

Although the adjusted R^2^ values are very low for predicting channel preferences (explaining between 0.4% and 6.3% of the variation in the data) and trust (explaining between 1.9% and 8.6% of variation in the data) using demographics, this fits with the results of a similar study using demographics to predict trust in sources on science issues [3]. Because the goal of this analysis was not to ultimately create a model to predict channel preference and trust, but rather to explore which demographics might have differences in preferences, the low R^2^ values are not a large concern. However, the low R^2^ values do illustrate that demographics are not the only variable driving communication preferences; in fact, there are clearly many other factors driving preferences that are beyond sociodemographics.

### Limitations and future research

There are limitations that need to be considered when interpreting these results. As stated upfront, this sample is biased towards males, older people, white people, and more highly educated people. Although gender and age were weighted to make the results more representative of the general public, education, race, and ethnicity could not be accounted for. However, since this paper also explored the impact of demographics on channels and trust preferences, it would be possible to speculate what numbers may be slightly impacted by having a sample that is more highly educated and white than the overall population and the directionality of these differences. For example, we know that the sample is more highly educated, and people with more education have higher preference for all sources. Therefore, if this sample had no education bias, the preference numbers may all be slightly lower. Additionally, those with less education were more likely to check the box that they do not look for information on nature-related topics, indicating again that people with education are more likely to search out information.

Future research could aim to better understand why people do or do not trust certain sources. Since demographics only account for a small portion of the variation in the data, it would be interesting to conduct qualitative interviews to better understand why people do or do not trust sources on nature-related topics. Additionally, while this study focused only on levels of trust for receiving nature-related information, it would be beneficial to know if people tend to have similar trust patterns among other topics. Likewise, it would be important to understand how degree of trust in a source–and ultimately message elaboration–could be influenced by factors other than the source itself (e.g., message content and framing).

This study investigated preferences for 10 different channels of information, but some channel categories were quite broad. Future studies should consider narrowing the categories even further, particularly for the channels we found to be highly preferred. For example, our category of “online communications” included e-newsletters and social media, but it would be insightful to see how preferences vary across different social media platforms in order to further tailor communication efforts. Additionally, it would be interesting to see if some information channels elicit more trust than others. This paper only looked at trust as it relates to sources, but the channels may also evoke different degrees of trust.

It would also be worthwhile to further investigate where people tend to get their information on nature-related topics, for both channels and sources. People do not always use their most preferred channels because other channels may have the information more easily accessible [4]. Additionally, it seems unlikely that the sources with the highest trust are what people actually tend to use. In fact, a survey of the Canadian public found that watching visual media and reading publications were the two most commonly utilized channels, and journalists/media and friends/family were the two most commonly utilized sources [42]. More research could help better understand discrepancies between communication preferences and where people actually obtain information. It would be helpful to know whether people would change to using their most preferred channels and sources if the information they are looking for is more readily available and accessible via their preferred communication means.

## Supporting information

S1 File The survey instrument. (PDF)

## References

[1] Cox R. Environmental communication and the public sphere 3rd ed. Thousand Oaks, CA: Sage publications; 2013.

[2] Tucker M, Napier TL. Determinants of perceived agricultural chemical risk in three watersheds in the Midwestern United States. Journal of Rural Studies. 2001;17(2):219–33. 10.1016/S0743-0167(00)00044-9

[3] Brewer PR, Ley BL. Whose Science Do You Believe? Explaining Trust in Sources of Scientific Information About the Environment. Science Communication. 2013;35(1):115–37. 10.1177/1075547012441691

[4] Mitchell A, Jeffrey G, Barthel M, Shearer E. The modern news consumer: News attitudes and practices in the digital era. Pew Research Center, 2016.

[5] Nguyen VM, Rudd MA, Hinch SG, Cooke SJ. Differences in Information Use and Preferences Among Recreational Salmon Anglers: Implications for Management Initiatives to Promote Responsible Fishing. Human Dimensions of Wildlife. 2012;17(4):248–56. 10.1080/10871209.2012.675412

[6] Rubin AM. Uses, Gratifications, and Media Effects Research In: Bryant J, Zillman D, editors. Perspectives on Media Effects. Hillsdale, NJ: Lawrence Erlbaum Associates; 1986 p. 281–301.

[7] Boellstorff DE, Borisova T, Smolen MD, Evans JM, Calabria J, Adams DC, et al Audience Preferences for Water Resource Information from Extension and Other Sources. Natural Sciences Education 2013;42(1):123.

[8] McCabe MB, Corona R, Weaver R. Sustainability for Hispanics in California: Do They Really Care? Global Journal of Business Research. 2013;7(2):103–12.

[9] DeYoung SE, Wachtendorf T, Farmer AK, Penta SC. NOAA Radios and Neighbourhood Networks: Demographic Factors for Channel Preference for Hurricane Evacuation Information. Journal of Contingencies and Crisis Management. 2016;24(4):275–85. 10.1111/1468-5973.12123

[10] Takahashi B, Tandoc EC. Media sources, credibility, and perceptions of science: Learning about how people learn about science. Public Understanding of Science. 2016;25(6):674–90. 10.1177/0963662515574986 25792288 10.1177/0963662515574986

[11] Lu L, Yuan YC. Shall I Google it or ask the competent villain down the hall? The moderating role of information need in information source selection. Journal of the American Society for Information Science and Technology. 2011;62(1):133–45. 10.1002/asi.21449

[12] Berlo DK. The Process of Communication: An Introduction to Thery and Practice. New York, NY: Rinehart Press; 1960.

[13] Shannon C, Weaver W. The mathematical theory of communication Urbana, IL: University of Illinois Press; 1949.

[14] O’Keefe DJ. Persuasion: Theory and research. Los Angeles: Sage; 2016.

[15] Metzger MJ, Flanagin AJ, Eyal K, Lemus DR, McCann RM. Credibility for the 21st century: Integrating perspectives on source, message, and media credibility in the contemporary media environment. Annals of the International Communication Association. 2003;27(1):293–335.

[16] Lewis JD, Weigert A. Trust as a social reality. Social forces. 1985;63(4):967–85.

[17] Johnson HH, Scileppi JA. Effects of ego-involvement conditions on attitude change to high and low credibility communicators. Journal of Personality and Social Psychology. 1969;13(1):31–6. 10.1037/h0027992 5352373 10.1037/h0027992

[18] Eagly AH, Chaiken S. The psychology of attitudes. Fort Worth: Harcourt Brace Jovanovich College Publishers; 1993.

[19] Petty RE, Cacioppo JT. Communication and persuasion: central and peripheral routes to attitude change New York: Springer-Verlag; 1986.

[20] Malka A, Krosnick JA, Langer G. The association of knowledge with concern about global warming: trusted information sources shape public thinking. Risk analysis: an official publication of the Society for Risk Analysis. 2009;29(5):633–47. 10.1111/j.1539-6924.2009.01220.x 19302280 10.1111/j.1539-6924.2009.01220.x

[21] Ongare D, Macharia A, Mwakaje A, Muchane M, Warui C, Mugoya C, et al Environmental Communication: A Review of Information Sources and Communication Channels for Enhanced Community-Based Natural Resource Management in the Greater Mara Region of Kenya. Journal of Education for Sustainable Development. 2013;7(1):65–74. 10.1177/0973408213495608

[22] Frewer L. The public and effective risk communication. Toxicology letters. 2004;149(1–3):391–7. 10.1016/j.toxlet.2003.12.049 15093286 10.1016/j.toxlet.2003.12.049

[23] Wachinger G, Renn O, Begg C, Kuhlicke C. The risk perception paradox—implications for governance and communication of natural hazards. Risk analysis. 2013;33(6):1049–65. 10.1111/j.1539-6924.2012.01942.x 23278120 10.1111/j.1539-6924.2012.01942.x

[24] Lee C-J, Scheufele DA, Lewenstein BV. Public Attitudes toward Emerging Technologies. Science Communication. 2005;27(2):240.

[25] Twenge JM, Campbell WK, Carter NT. Declines in Trust in Others and Confidence in Institutions Among American Adults and Late Adolescents, 1972–2012. Psychological Science. 2014;25(10):1914–23. 10.1177/0956797614545133 25205784 10.1177/0956797614545133

[26] Chanley VA, Rudolph TJ, Rahn WM. The Origins and Consequences of Public Trust in Government: A Time Series Analysis. The Public Opinion Quarterly. 2000;64(3):239–56. 11114267 10.1086/317987

[27] Flickinger RS, Rhine SL, Bennett SE, Bennett LLM. "Video Malaise" Revisited: Public Trust in the Media and Government. The Harvard International Journal of Press/Politics. 1999;4(4):8–23. 10.1177/1081180X9900400402

[28] Hetherington MJ. The Political Relevance of Political Trust. The American Political Science Review. 1998;92(4):791–808. 10.2307/2586304

[29] Delhey J, Newton K. Predicting Cross-National Levels of Social Trust: Global Pattern or Nordic Exceptionalism? European Sociological Review. 2005;21(4):311–27. 10.1093/esr/jci022

[30] McCaffrey SM, Olsen CS. Research perspectives on the public and fire management: A synthesis of current social science on eight essential questions. U.S. Department of Agriculture, 2012.

[31] Ferguson E, Spence A, Townsend E, Prowse C, Palmer J, Fleming P, et al What type of information is trusted by whom? A multilevel analysis of the stability of the information source-trust association for blood transfusion. Transfusion. 2009;49(8):1637–48. 10.1111/j.1537-2995.2009.02179.x 19392778 10.1111/j.1537-2995.2009.02179.x

[32] Brossard D, Nisbet MC. Deference to scientific authority among a low information public: understanding U.S opinion on agricultural biotechnolgy. International Journal of Public Opinion Research. 2007;19(1):24.

[33] Brossard D, Shanahan J. Do Citizens Want to Have Their Say? Media, Agricultural Biotechnology, and Authoritarian Views of Democratic Processes in Science. Mass Communication and Society. 2003;6(3):291–312. 10.1207/S15327825MCS0603_4

[34] Liu H, Priest S. Understanding public support for stem cell research: media communication, interpersonal communication and trust in key actors. Public Understanding of Science. 2009;18(6):704–18. 10.1177/0963662508097625

[35] Nisbet MC, Goidel RK. Understanding citizen perceptions of science controversy: bridging the ethnographic—survey research divide. Public Understanding of Science. 2007;16(4):421–40. 10.1177/0963662506065558

[36] Gauchat G. Politicization of Science in the Public Sphere: A Study of Public Trust in the United States, 1974 to 2010. American Sociological Review. 2012;77(2):167–87. 10.1177/0003122412438225

[37] Kellstedt PM, Zahran S, Vedlitz A. Personal Efficacy, the Information Environment, and Attitudes Toward Global Warming and Climate Change in the United States. Risk Analysis. 2008;28(1):113–26. 10.1111/j.1539-6924.2008.01010.x 18304110 10.1111/j.1539-6924.2008.01010.x

[38] Nisbet MC, Myers T. The Polls Trends: Twenty Years of Public Opinion about Global Warming. Public Opinion Quarterly. 2007;71(3):444–70. 10.1093/poq/nfm031

[39] Bickerstaff K, Lorenzoni I, Pidgeon NF, Poortinga W, Simmons P. Reframing nuclear power in the UK energy debate: nuclear power, climate change mitigation and radioactive waste. Public Understanding of Science. 2008;17(2):145–69. 10.1177/0963662506066719 19391376 10.1177/0963662506066719

[40] Yoo K-H, Gretzel U, Zanker M. Persuasive recommender systems: conceptual background and implications New York, NY: Springer Science & Business Media; 2012.

[41] Theng Y-L, Goh LYQ, Tin MT, Sopra R, Kumar SKP, editors. Trust cues fostering initial consumers' trust: usability inspection of nutrition and healthcare websites. Proceedings of the 2nd ACM SIGHIT International Health Informatics Symposium; 2012: ACM.

[42] Federal, Provincial, and Territorial Governments of Canada. Canadian Nature Survey: Awareness, participation, and expenditures in nature-based recreation, conservation, and subsistence activities. Ottawa, ON: 2014.

[43] Dillman DA, Smyth JD, Christian LM. Internet, Phone, Mail, and Mixed-Mode Surveys: The Tailored Design Method. US: John Wiley & Sons Inc; 2014.

[44] Wilkins EJ, Miller HM, au>Schuster R. Results of a U.S. General Public Survey to Inform the 2018 North American Waterfowl Management Plan Update. In: Survey USG, editor. 2017.

[45] Tucker M, Napier TL. Preferred sources and channels of soil and water conservation information among farmers in three midwestern US watersheds. Agriculture, Ecosystems and Environment. 2002;92(2):297–313. 10.1016/S0167-8809(01)00293-6

[46] Eisinga R, Te Grotenhuis M, Pelzer B. The reliability of a two-item scale: Pearson, Cronbach, or Spearman-Brown? International journal of public health. 2013;58(4):637–42. 10.1007/s00038-012-0416-3 23089674 10.1007/s00038-012-0416-3

[47] Vaske JJ. Survey research and analysis: applications in parks, recreation and human dimensions. State College, Pa: Venture Publishing; 2008.

[48] Kalton G, Flores-Cervantes I. Weighting Methods. Journal of Official Statistics. 2003;19(2):81–97.

[49] MacDonald SE, Newburn‐Cook CV, Schopflocher D, Richter S. Addressing nonresponse bias in postal surveys. Public Health Nursing. 2009;26(1):95–105. 10.1111/j.1525-1446.2008.00758.x 19154197 10.1111/j.1525-1446.2008.00758.x

[50] Nisbet MC, Myers T. The polls—trends twenty years of public opinion about global warming. Public Opinion Quarterly. 2007;71(3):444–70.

