# Supplementary material for: Communicating information on nature-related topics: Preferred information channels and trust in sources
Source: PLoS One. 2018 Dec 12;13(12):e0209013. doi: 10.1371/journal.pone.0209013 (PMC6291159; doi:10.1371/journal.pone.0209013)
Supplement: S1 File — (PDF) [file pone.0209013.s001.pdf]

In this survey, we'll be asking for your opinions about and interactions with nature, specifically wetlands. **Wetlands include swamps, marshes, bogs, shallow ponds (less than 6 feet deep), and shallow areas on lakeshores and seashores**. Some wetlands are only wet some of the year, while others are wet year round. They can be in cities or in rural areas and can be smaller than a basketball court or cover several square miles. **We'll also be asking questions about waterfowl, such as ducks and geese,** which rely on the resources wetlands provide. ***While you may not have ever heard of wetlands or waterfowl before, we still want to hear from you!***

## SECTION A: NATURE AND WETLANDS ACTIVITIES

**1.** In the **last 12 months**, (A) Which of the following activities related to nature and wetlands did you participate in, if any? and (B) In the future, which activities are you interested in participating in, regardless of whether you have done them before? *In the first column, please check Yes or No for each activity. Then circle the number which best represents your interest level in this activity.*

| Activity | A. In the last 12 months, I participated in this activity. | | B. How likely are you to participate in this activity in the next 12 months? | | |
|---|---|---|---|---|---|
| | | | Not at all likely | Somewhat likely | Very likely |
| Backyard/at-home nature activities (e.g., gardening, landscaping) | ❒ Yes | ❒ No | 0 | 1 | 2 |
| Spending time in nature away from home (e.g., picnicking) | ❒ Yes | ❒ No | 0 | 1 | 2 |
| Viewing/feeding/photographing birds | ❒ Yes | ❒ No | 0 | 1 | 2 |
| Viewing/photographing other types of wildlife | ❒ Yes | ❒ No | 0 | 1 | 2 |
| Fishing (salt or freshwater) | ❒ Yes | ❒ No | 0 | 1 | 2 |
| Hunting waterfowl (e.g., ducks, geese) | ❒ Yes | ❒ No | 0 | 1 | 2 |
| Hunting all other game (e.g., deer, rabbit) | ❒ Yes | ❒ No | 0 | 1 | 2 |
| Non-motorized outdoor recreation activities (e.g., hiking, camping, horseback riding, bicycling, canoeing) | ❒ Yes | ❒ No | 0 | 1 | 2 |
| Motorized outdoor recreation activities (e.g., motorized boating, riding ATVs, snowmobiling) | ❒ Yes | ❒ No | 0 | 1 | 2 |
| Learning about nature (e.g., reading/ watching videos about nature, attending nature festivals/lectures) | ❒ Yes | ❒ No | 0 | 1 | 2 |
| Other *(please specify)* _____________ | ❒ Yes | ❒ No | 0 | 1 | 2 |

Next, we would like to know more about how you feel about hunting and birdwatching, even if you don't participate in these activities.

**2.** Using the scales below, please complete the following statement: "For me, **hunting** in the next 12 months would be..." *Please circle one number for each row.*

| | Very | Somewhat | Neither | Somewhat | Very | |
|---|---|---|---|---|---|---|
| **Unpleasant** | 1 | 2 | 3 | 4 | 5 | **Pleasant** |
| **Boring** | 1 | 2 | 3 | 4 | 5 | **Interesting** |

**3.** Using the scales below, please complete the following statement: "For me, **birdwatching** in the next 12 months would be…" *Please circle one number for each row.*

| | Very | Somewhat | Neither | Somewhat | Very | |
|---|---|---|---|---|---|---|
| **Unpleasant** | 1 | 2 | 3 | 4 | 5 | **Pleasant** |
| **Boring** | 1 | 2 | 3 | 4 | 5 | **Interesting** |

**4.** How much do you agree or disagree with the following statements? *Please circle one number for each statement.*

| | Strongly disagree | Disagree | Neither agree nor disagree | Agree | Strongly agree | Don't know |
|---|---|---|---|---|---|---|
| People important to me would support my **hunting** in the next 12 months. | 1 | 2 | 3 | 4 | 5 | DK |
| If I wanted to, I could easily go **hunting** in the next 12 months. | 1 | 2 | 3 | 4 | 5 | DK |
| People important to me would support my **birdwatching** in the next 12 months. | 1 | 2 | 3 | 4 | 5 | DK |
| If I wanted to, I could easily go **birdwatching** in the next 12 months. | 1 | 2 | 3 | 4 | 5 | DK |

**5.** What would prevent you from **hunting** in the next 12 months? *Write your answer or check the box below.*

______________________________________________________________________

______________________________________________________________________

❒ I don't know/I've never thought about it

**6.** What would prevent you from **birdwatching** in the next 12 months? *Write your answer or check the box below.*

______________________________________________________________________

______________________________________________________________________

❒ I don't know/I've never thought about it

**7.** We're interested in whether you know people who participate in certain kinds of nature-related activities. Do you know any of the following types of people? *Please check all that apply for each type of person OR check "No one" if you do not know that type of person.*

| Type of person | Acquaintance | Close Friend | Relative | No one |
|---|---|---|---|---|
| Hunter | ❒ | ❒ | ❒ | ❒ |
| Birdwatcher | ❒ | ❒ | ❒ | ❒ |
| Wildlife photographer | ❒ | ❒ | ❒ | ❒ |
| Conservationist | ❒ | ❒ | ❒ | ❒ |

**8.** What types of wild birds do you or would you prefer to see? *Please circle one number for each type of bird.*

| Type of bird | Not at all preferred | Slightly preferred | Somewhat preferred | Very preferred | Don't know |
|---|---|---|---|---|---|
| Waterfowl (ducks, geese, etc.) | 0 | 1 | 2 | 3 | DK |
| Other game birds (grouse, pheasant, turkey, etc.) | 0 | 1 | 2 | 3 | DK |
| Hummingbirds | 0 | 1 | 2 | 3 | DK |
| Water birds (shorebirds, herons, etc.) | 0 | 1 | 2 | 3 | DK |
| Birds of prey (hawks, eagles, owls, etc.) | 0 | 1 | 2 | 3 | DK |
| Songbirds (warblers, sparrows, thrushes, finches, etc.) | 0 | 1 | 2 | 3 | DK |
| Other birds (anything not mentioned) | 0 | 1 | 2 | 3 | DK |

**9.** Please indicate your level of involvement in the following **conservation and wildlife-related** activities in the **last 12 months**. *Please circle one number for each activity.*

| Activity | Never | Rarely | Sometimes | Often | Very often |
|---|---|---|---|---|---|
| Made my yard or my land more desirable to wildlife | 0 | 1 | 2 | 3 | 4 |
| Volunteered to improve wildlife habitat in my community | 0 | 1 | 2 | 3 | 4 |
| Talked to others in my community about conservation issues | 0 | 1 | 2 | 3 | 4 |
| Participated as an active member in a nature, outdoor, or conservation group | 0 | 1 | 2 | 3 | 4 |
| Donated money to support wildlife/habitat conservation | 0 | 1 | 2 | 3 | 4 |

**10.** Please indicate your level of involvement in the following **wetlands/waterfowl conservation** activities in the **last 12 months**. *Please circle one number for each activity.*

| Activity | Never | Rarely | Sometimes | Often | Very often |
|---|---|---|---|---|---|
| Worked on land improvement projects related to wetlands/waterfowl conservation | 0 | 1 | 2 | 3 | 4 |
| Attended meetings about wetlands/waterfowl conservation | 0 | 1 | 2 | 3 | 4 |
| Volunteered my personal time and effort to conserve wetlands/waterfowl | 0 | 1 | 2 | 3 | 4 |
| Contacted elected officials or government agencies about wetlands/waterfowl conservation | 0 | 1 | 2 | 3 | 4 |
| Voted for candidates or ballot issues to support wetlands/waterfowl conservation | 0 | 1 | 2 | 3 | 4 |
| Advocated for political action to conserve wetlands/waterfowl | 0 | 1 | 2 | 3 | 4 |

## SECTION B: SOURCES OF INFORMATION ABOUT CONSERVATION ISSUES

**11.** When you are looking for information about nature-related topics, such as recreational activities, wildlife, natural areas, or conservation issues, in which of the following ways do you prefer to get this information? *Please circle only one number for each source OR check "I do not look for information about conservation issues" at the bottom of the table.*

| | Not at all preferred | Slightly preferred | Somewhat preferred | Very preferred |
|---|---|---|---|---|
| Receive or follow **online** communications (email updates or newsletters, social media, etc.) | 0 | 1 | 2 | 3 |
| Read or access **online** content (websites, apps, blogs, magazines, newspapers, books, reports, etc.) | 0 | 1 | 2 | 3 |
| Read **printed** publications (magazines, newspapers, books, reports, newsletters, brochures, etc.) | 0 | 1 | 2 | 3 |
| Watch visual media **online** (videos, webinars, television shows, movies, etc.) | 0 | 1 | 2 | 3 |
| Watch visual media through cable, satellite, or network (television shows, movies, etc.) | 0 | 1 | 2 | 3 |
| Listen to **recorded** audio media (podcasts, audio books, etc.) | 0 | 1 | 2 | 3 |
| Listen to **live** audio media (radio, etc.) | 0 | 1 | 2 | 3 |
| Talk with other people about nature-related topics (friends, family, colleagues, etc.) | 0 | 1 | 2 | 3 |
| Through personal experience | 0 | 1 | 2 | 3 |
| Attend educational opportunities (courses, seminars, conferences, etc.) | 0 | 1 | 2 | 3 |
| Other *(please specify)* ____________ ______________________________ | 0 | 1 | 2 | 3 |

❒ **I do not look for information about nature-related topics *(skip to Section C, question 13)***

**12.** When you are looking for information about nature-related topics, such as recreational activities, wildlife, natural areas or conservation issues, how much do you trust the following sources to provide accurate information? *Please circle only one number for each source.*

| | Do not trust at all | Trust a little | Trust somewhat | Trust a lot | Trust completely |
|---|---|---|---|---|---|
| Federal government | 0 | 1 | 2 | 3 | 4 |
| State government | 0 | 1 | 2 | 3 | 4 |
| Local government (city, county, etc.) | 0 | 1 | 2 | 3 | 4 |
| Conservation groups | 0 | 1 | 2 | 3 | 4 |
| Universities/Educational organizations | 0 | 1 | 2 | 3 | 4 |
| National media/news | 0 | 1 | 2 | 3 | 4 |
| Local media/news | 0 | 1 | 2 | 3 | 4 |
| Friends, family, neighbors, colleagues | 0 | 1 | 2 | 3 | 4 |
| Scientific organizations | 0 | 1 | 2 | 3 | 4 |
| Religious organizations | 0 | 1 | 2 | 3 | 4 |
| Other *(please specify)* _____________ | 0 | 1 | 2 | 3 | 4 |

**SECTION C: YOUR OPINIONS ABOUT WETLANDS**

**13.** Wetlands include swamps, marshes, bogs, shallow ponds (less than 6 feet deep), and shallow areas on lakeshores and seashores. Some wetlands are only wet some of the year, while others are wet year round. They can be in cities or in rural areas and can be smaller than a basketball court or cover several square miles. Based on this description, do you know of any wetlands in your local area or community? *Please check only one.*

❒ Yes

❒ No

❒ Don't know

**14.** Have you visited any wetlands in the last 12 months? *Please check only one.*

❒ Yes → *Go to next question.*

❒ No → *Skip to question #16.*

**15.** What was the purpose of your wetlands visit(s)? *Please check all that apply.*

- ❒ Enjoying nature/picnicking/nature photography
- ❒ Walking/dog walking/hiking/biking
- ❒ Boating
- ❒ Wildlife viewing/birdwatching/wildlife photography
- ❒ Fishing
- ❒ Hunting
- ❒ Other (please specify ___________________________

**16.** Wetlands perform a variety of functions which are beneficial to people. When wetlands are lost or degraded, these benefits can be reduced or disappear altogether. How concerned would you be if the following benefits were substantially reduced in your community due to a loss of wetlands? *Please circle one number for each benefit.*

| Benefit | Not at all concerned | Slightly concerned | Somewhat concerned | Very concerned |
|---|---|---|---|---|
| **A.** Flooding protection | 0 | 1 | 2 | 3 |
| **B.** Erosion protection | 0 | 1 | 2 | 3 |
| **C.** Wildlife viewing and birdwatching | 0 | 1 | 2 | 3 |
| **D.** Hunting opportunities | 0 | 1 | 2 | 3 |
| **E.** Storage of greenhouse gases, such as carbon | 0 | 1 | 2 | 3 |
| **F.** Clean water | 0 | 1 | 2 | 3 |
| **G.** Clean air | 0 | 1 | 2 | 3 |
| **H.** Providing a home for wildlife | 0 | 1 | 2 | 3 |
| **I.** Providing a home for animals such as butterflies and bees that pollinate plants and crops | 0 | 1 | 2 | 3 |
| **J.** Scenic places for inspiration or spiritual renewal | 0 | 1 | 2 | 3 |

**17.** Which of the wetlands benefits listed above would you be **most and least** concerned about being substantially reduced in your community? *Please write the letter associated with the benefit you are most concerned about losing and then the letter associated with the benefit you are least concerned about losing on the lines below. Use a letter only one time.*

| Benefit **most** concerned about _____ | Benefit l**east** concerned about _____ |
|---|---|

## SECTION D: ABOUT YOU

To help us compare your responses to those of others, we have some questions about you. Please be assured that all of your answers will remain completely confidential.

**18.** In what year were you born? 19____

**19.** Are you…?

- ❒ Male
- ❒ Female

**20.** What is the highest grade (or year) of regular school you have completed? *Please check only one.*

- ❒ Some high school or less
- ❒ High school diploma or GED
- ❒ Some college (no degree)
- ❒ Associate's degree (2 years)
- ❒ Bachelor's degree (4 years)
- ❒ Graduate or professional school

**21.** Which of these categories best describes the place where you live now and where you lived during most of the time you were growing up (that is, until age 16)? *Please check only one in each column.*

| **Where you live now** | **Where you grew up** |
|---|---|
| ❒ Large urban area (population of 500,000 or more) | ❒ Large urban area (population of 500,000 or more) |
| ❒ Medium urban area (population between 50,000 and 500,000) | ❒ Medium urban area (population between 50,000 and 500,000) |
| ❒ Small city (population between 10,000 and 50,000) | ❒ Small city (population between 10,000 and 50,000) |
| ❒ Small town (population between 2,500 and 10,000) | ❒ Small town (population between 2,500 and 10,000) |
| ❒ Rural area (population less than 2,500) | ❒ Rural area (population less than 2,500) |

**22.** Is a nature-related profession (such as fisheries, forestry, farming, environmental science, or conservation) the primary source of your personal income? *Please check only one.*

- ❒ Yes
- ❒ No

**23.** What ethnicity do you consider yourself? *Please check only one.*

- ❒ Hispanic or Latino
- ❒ Not Hispanic or Latino

**24.** From what racial origin(s) do you consider yourself? *Please check all that apply.*

- ❒ American Indian or Alaskan Native
- ❒ Asian
- ❒ Black or African American
- ❒ Native Hawaiian or other Pacific Islander
- ❒ White

# Thank you!

**Comments? Please write them below.**

PAPERWORK REDUCTION ACT STATEMENT: The Paperwork Reduction Act requires us to tell you why we are collecting this information, how we will use it, and whether or not you have to respond. The information that we collect in this survey will help us understand the general public's experiences with and views on nature and wetlands and help the U.S. Fish and Wildlife Service and state agencies manage waterfowl and wetlands through the North American Waterfowl Management Plan. Your response is voluntary. An agency may not conduct or sponsor a collection of information unless it displays a valid OMB Control Number. We estimate it will take an average of 20 minutes to complete this survey. You may send comments concerning the burden estimate or any aspect of the survey to the Information Collection Clearance Officer, U.S. Geological Survey, 12201 Sunrise Valley Dr. 807, Reston, VA 20192. OMB CONTROL #1028-0120. EXPIRATION DATE 11/30/2019.
